# Influence of Combinations of Chemical and Organic Fertilizers on Biochemical Responses of Tomato Fruits in Different Maturity Stages

**DOI:** 10.1021/acsomega.5c02531

**Published:** 2025-07-14

**Authors:** Aytekin EKINCIALP, Selma KIPCAK BITIK, Çeknas ERDINÇ, Suat SENSOY

**Affiliations:** † Van Yuzuncu Yil University, Baskale Vocational School, 65080 Van, Türkiye; ‡ Van Yuzuncu Yil University, Faculty of Agriculture, Department of Agricultural Biotechnology, 65080 Van, Türkiye; § Van Yuzuncu Yil University, Faculty of Agriculture, Department of Horticulture, 65080 Van, Türkiye

## Abstract

This study evaluates the impact of organic and conventional fertilizers on the quality attributes of Nergis F1 tomatoes. The fertilizers tested included full-dose vermicompost (VC), organo-mineral fertilizer (OMF, 11:11:11), chemical fertilizer (CF, 20:20:20), and their combinations. Key quality parameters were assessed, including pH, titratable acidity (TA), total soluble solids (TSS), ascorbic acid (AsA), lycopene, β-carotene, organic acids, phenolic compounds, total antioxidant capacity (TAC), total phenols (TP), respiration rate, and ethylene production. Results showed that the OMF treatment significantly increased TSS at the red maturity stage (8.33 °Brix), while the highest TSS value (8.65 °Brix) was observed in T1, followed closely by T4. Treatments T10 and T9 also exhibited elevated TSS levels at the red and pink maturity stages, respectively. Additionally, combinations of OMF and VC positively influenced organic acid and phenolic compound concentrations. These findings highlight the potential of organic and organo-mineral fertilizers to enhance tomato quality and support sustainable agricultural practices.

## Graphical abstract

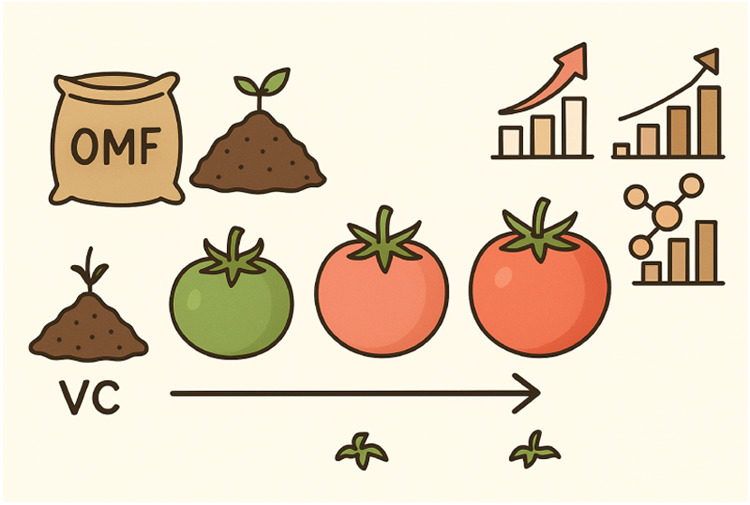

## 1 Introduction

Tomatoes (*Solanum lycopersicum* L.) are a vital crop valued for their rich content of bioactive compounds such as lycopene, β-carotene, vitamin C, and polyphenols. Consumed fresh and widely processed into products like sauces, juices, and ketchup, global tomato production reaches approximately186 million tons annually, with China, India, and Türkiye as leading producers.^1^ However, conventional agriculture’s reliance on chemical fertilizers and pesticides poses environmental and health risks, including biodiversity loss, pollution, and pest resistance. Prolonged use of mineral fertilizers, while enhancing productivity, can degrade soil fertilityand microbial populations, and contributes to erosion, acidification, and CO_2_ emissions.^2,3^ With global food demand rising alongside population growth, sustainable agricultural practices are crucial forbalancing productivity with environmental protection.^4,5^

Organic alternatives such as vermicompost, which is rich in beneficial microorganisms and nutrients, can improve soil properties and promote plant growth.^6,7^ Similarly, organo-mineral fertilizers blending mineral inputs with organic enhance soil health, nutrient uptake, and productivity while reducing soil degradation.^8,9^ Studies have highlight the positive impact of these fertilizers on tomato yield and quality.^10,11^

Tomatoes are also rich in secondary metabolites such as polyphenols, carotenoids, and flavonoids, which influence fruit quality and offer health benefits, including reduced risks of cardiovascular diseases, cancer, and neurodegenerative disorders.^12,13^ Sustainable practices utilizing organic and organo-mineral fertilizers provide an environmentally friendly approach to meeting future agricultural demands.

In recent years, integrated fertilization strategies combining chemical fertilizers (CF), organo-mineral fertilizers (OMF), and vermicompost (VC) have gained attention due to their synergistic effects on crop productivity, fruit quality, and soil health.^14–16^ In this context, the present study employed combinations of CF, OMF, and VC at varying proportions to simulate practical fertilization gradients commonly tested in open-field production systems. These ratios were selected not only based on their agronomic feasibility and relevance to current horticultural practices, but also to allow assessment of dose-dependent physiological and biochemical responses in tomato fruits under field conditions. By adopting this integrated approach, the study aims to provide a comparative understanding of how different fertilization regimes influence nutritional quality and ripening dynamics across fruit maturity stages.

Our hypothesis is that the use of organic and organo-mineral fertilizers, especially in combination, can improve the nutritional quality and biochemical profile of Nergis F1 tomatoes, leading to higher levels of total soluble solids (TSS), ascorbic acid (AsA), lycopene, β-carotene, organic acids, and phenolic compounds compared to conventional chemical fertilizers. This could contribute to more sustainable and health-conscious agricultural practices.

## 2 Materials and Methods

### 2.1 Experimental Setup

The experimental design utilized seedlings of the Nergis F1 (Manier Seed) tomato variety as the primary material. The study encompassed 30 plots arranged in accordance with a randomized complete block design, comprising three replications. A 1-m gap separated individual plots, while a 2-m distance was maintained between blocks. The experiment was conducted in open-field plots located at Van Yüzüncü Yıl University (38° 34′ 25′ North latitude and 43° 17′ 40″ East longitude) were used. Tomato seedlings were transplanted on June 11, 2021, with a spacing of 100 cm between rows and 50 cm between plants within rows. Consequently, a total of 600 tomato seedlings were planted, with 20 plants allocated to each plot. To account for edge effects, sampling was performed on six centrally positioned plants within each plot. These six plants were permanently tagged at the early flowering stage to ensure consistency across all maturity stages. Fruit sampling was carried out at green, pink (medium ripe/half ripe), and red maturity stages. At each stage, fruits were harvested from the middle canopy level of each tagged plant to reduce within-plant variability related to light or position. Fruits were selected based on uniformity in size, maturity, and the absence of physical defects. This protocol ensured a standardized and unbiased sampling process for all biochemical and quality analyses. The soil characteristics of the experimental area were analyzed prior to planting, indicating nonsaline conditions (0.01% EC), low organic matter content (0.93%), high lime content (10.84%), and a slightly alkaline pH (7.83). Throughout the growing period, seedlings were irrigated using a drip irrigation system to maintain consistent soil moisture. Detailed soil properties and climatic conditions for the 2021 growing season are presented in Table 1.

**Table 1:** Physicochemical Properties of the Experimental Soil and Climatic Data during the 2021 Growing Season

parameter	value	parameter	May	June	July	August	September
*P* (mg kg^–1^)	5.50	average temperature (°C)	19.2	21.1	24.5	26.4	22.8
*K* (mg kg^–1^)	11.04	minimum temperature (°C)	10.5	14.3	17.1	19.6	16.5
organic matter (%)	0.93	maximum temperature (°C)	27.8	27.9	31.8	33.2	29.1
lime (%)	10.84	sunshine duration (h)	11.9	12.7	12.5	12.6	11.8
pH (%)	7.83	precipitation (mm)	8.1	14.7	12.7	15.5	1.1
electrical conductivity/salt (%)	0.01	wind (m s^–1^)	3.1	2.7	2.1	2.3	2.1
structure (%)	sand: 37, clay: 24.3, silt: 38.6	relative humidity (%)	41.8	48.3	34.2	30.9	35.8
N (%)	0.17	evaporation (mm)	65.2	188.5	199.4	213.4	25.0
Mn (mg kg^–1^)	0.98						
Na (mg kg^–1^)	86.35						
Fe (mg kg^–1^)	1.12						
Cu (mg kg^–1^)	0.53						
Zn (mg kg^–1^)	0.46						
Ca (mg kg^–1^)	1025						
Mg (mg kg^–1^)	134						

### 2.2 Fertilization Program

These combinations were designed to reflect common agricultural practices aimed at optimizing nutrient supply while enhancing soil health. The varying proportions of CF, OMF, and VC were selected based on their complementary nutrient profiles, aiming to balance immediate nutrient availability (from chemical fertilizers) with long-term improvements in soil organic matter and microbial activity (from vermicompost and organo-mineral fertilizers). This strategy aligns with sustainable agriculture goals, addressing nutrient-use efficiency and minimizing environmental impact, especially in regions facing soil degradation and input cost challenges.The experiment utilized three primary fertilizer sources: chemical fertilizer (CF) provided by Gübretaş, vermicompost (VC) from Yaşa Tarım, and organo-mineral fertilizer (OMF) by Hektaş (Table 2). These fertilizers were combined in varying proportions to create ten distinct treatment groups, as delineated in Table 3. During the growing season, urea was applied twice at intervals of about 4 weeks to supplement nitrogen levels in the plots where chemical fertilizer was applied alone or in combination.

**Table 2:** Contents of Fertilizers Used in the Plant Growing Process

chemical fertilizer (CF) (20:20:20) (%)	triple super phosphate (TSP)	%46 nitrogen fertilizer	organo-mineral fertilizer (OMF) 11:11:11 (%)	vermicompost (VC) (%)
●total nitrogen: 20	●water-soluble phosphorus pentoxide: 39	●urea: 46	●organic matter: 18.00	●organic matter: 35.00
●ammonium nitrogen: 3.9	●phosphorus pentoxide soluble in neutral ammonium citrate (P_2_O_5_): 42		●total nitrogen: 11.00	●total nitrogen: 2.00
●nitrate nitrogen: 5.9			●ammonium nitrogen: 5.00	●organic nitrogen: 1.00
●urea (N-NH_2_): 10.2			●urea nitrogen: 6.00	●water-soluble potassium okxide: 2.00
●water-soluble phosphorus pentoxide: 20			●water-soluble potassium oxide: 11.00	●total phosphorus pentoxide: 0.40
●water-soluble potassium oxide: 20			●total phosphorus pentoxide: 11.00	●total humic acid + fulvic acid: 20.00
●water-soluble iron: 0.05			● water-soluble phosphorus pentoxide: 9.00	●C/N: 10.04
●water-soluble copper: 0.02			●water-soluble sulfur trioxide: 7.00	●Max. EC (dS m^–1^): 2.00
●water-soluble zinc: 0.02			●total sulfur trioxide: 11.00	●Max moisture: 35.00
●water-soluble manganese: 0.02			●total zinc: 0.10	●pH: 6–8
			●total humic acid + fulvic acid: 5.00	

**Table 3:** Doses of Fertilizers Used in the Plant Growing Process^a^

treatment	fertilizer combinations	fertilizer doses
T1	Control	-
T2	CF_(%100)_	19.8 kg da^–1^ N:P:K (20:20:20) + 14.21 kg da^–1^ TSP + 23.63 kg da^–1^ %46 urea
T3	VC_(%100)_	741.5 kg da^–1^
T4	OMF_(%100)_	134.82 kg da^–1^
T5	CF_(%25)_ + VC_(%75)_	4.75 kg da^–1^ N:P:K (20:20:20) + 3.55 kg da^–1^ TSP + 5.91 kg da^–1^ %46 urea +556.13 kg da^–1^ VC
T6	CF_(%50)_ + VC_(%50)_	9.9 kg da^–1^ N:P:K (20:20:20) + 7.11 kg da^–1^ TSP + 11.82 kg da^–1^ %46 urea +370.75 kg da^–1^ VC
T7	CF_(%75)_ + VC_(%25)_	14.85 kg da^–1^ N:P:K (20:20:20) + 10.66 kg da^–1^ TSP + 17.73 kg da^–1^ %46 urea +185.38 kg da^–1^ VC
T8	OMF_(%25)_ + VC_(%75)_	33.71 kg da^–1^ OMF + 556.13 kg da^–1^ VC
T9	OMF_(%50)_ + VC_(%50)_	67.41 kg da^–1^ OMF + 370.75 kg da^–1^ VC
T10	OMF_(%75)_ + VC_(%25)_	101.12 kg da^–1^ OMF + 185.38 kg da^–1^ VC

a CF: Chemical fertilizer; VC: Vermicompost; OMF: Organo-mineral fertilizer.

### 2.3 Fruit Quality Analyses

Upon washing and drying four fruits at the green maturity stage and six fruits at the pink and red maturity stages from each plot, the samples were homogenized using a blender for approximately 60 s. The following parameters were then determined:

*Total Soluble Solids (TSS)*: TSS content was measured using a digital refractometer (Atago, Tokyo, Japan), and results were expressed as °Brix per 100 g fresh weight.

*pH*: The pH of the fruits smples was determined using a pH meter (Thermo Fisher Scientific, USA).

*Titratable Acidity (TA)*: TA was measured by titrating the fruit juice with 0.1 N NaOH until pH reached 8.1, and the results were expressed as % citric acid.^17^

*Maturity Index (MI) and Flavor Index (FI)*: MI and FI were calculated using eqs 1 and 2, respectively.^18–20^

1 MI=TSS/TA

2 $$\mathrm{FI}=\mathrm{TA}+\left[\frac{\mathrm{TSS}}{20\times \mathrm{TA}}\right]$$

### 2.4 Ascorbic Acid (AsA) Analysis

For ascorbic acid (AsA) analysis, 3 g of pureed tomato samples were extracted with 5 mL of 3% metaphosphoric acid and centrifuged at 6500*g* for 10 min at +4 °C. The supernatant (0.5 mL) was diluted to 10 mL with 2.5% metaphosphoric acid, filtered through a 0.45 μm membrane, and analyzed using a C18 column (Phenomenex Luna, 250 × 4.60 mm, 5 μm)­with a DAD detector at 254 nm. l-ascorbic acid standards (50–2000 ppm) were used for quantification.^17^

### 2.5 Carotenoids (Lycopene and β-Carotene)

Fresh tomato tissue (25–50 g) was homogenized with an equal volume of distilled water. Approximately 0.4–0.6 g of the homogenate was mixed with 20 mL of a solution containing 0.05% BHT in acetone, 95% ethanol, and hexane (5:5:10 v/v) in amber vials. The vials were sealed, shaken at 180 rpm in crushed ice for 15 min, then mixed with 3 mL of deionized water and shaken for an additional 5 min. After phase separation, the hexane layer was collected for analysis. Lycopene content was determined at 503 nm using the formula:  μg = *A*_503_ * 31.3/sample weight (g).

For β-carotene, the hexane layer was analyzed at 452 nm using the following formula:  μg = *A*_452_ * 13.9/sample weight (g).^17,21,22^

### 2.6 Organic Acids Assay by HPLC-DAD

Organic acids were quantified using a modified method by Bevilacqua and Califano.^23^ Four fresh tomatoes were homogenized into puree, and 1 g of puree was mixed with 20 mL of 0.009 N sulfuric acid. The mixture was homogenized, shaken for 1 h, and centrifuged at 15,000 rpm for 15 min. The aqueous phase was filtered through coarse paper, a 0.45 μm membrane filter, and a SEP-PAK C18 cartridge. Analysis was performed using HPLC (Agilent 1100 series) equipped with an Aminex HPX-87 H column and DAD detection at 214 and 280 nm. A 0.009 N H_2_SO_4_ mobile phase was used and concentrations were determined by comparison with external standards.

### 2.7 Phenolic Compounds Assay by HPLC-DAD

Phenolic compounds were analyzed following the method of Rodriguez-Delgado et al.^24^ Tomato juice was extracted, centrifuged at 15,000 rpm for 15 min, and filtered through 0.45 μm Millipore filters. The filtrate was transferred to amber vials and analyzed by HPLC equipped with a DAD detector and a 250 × 4.6 mm, 4 μm ODS column (HiChrom, USA). A gradient mobile phase of Solvent A (methanol–acetic acid–water, 10:2:88) and Solvent B (methanol–acetic acid–water, 90:2:8) was used, with detection at 254 and 280 nm. The flow rate was 1 mL min^–1^, and 20 μL of sample was injected, enabling efficient separation and quantification of phenolic compounds.

### 2.8 Total Antioxidant Capacity (TAC) and Total Phenol (TP)

Total antioxidant capacity (TAC) and total phenol (TP) content were determined using the methods of Swain and Hillis^25^ for TP and Benzie and Strain^26^ for TAC. For TP, 150 μL of tomato sample was mixed with Folin-Ciocalteu reagent and sodium carbonate, incubated for 60 min, and measured at 760 nm. Results were expressed as gallic acid equivalents (GAE) per 100 mg FW. For TAC, the FRAP method was used: 150 μL of sample was mixed with FRAP reagent, incubated for 30 min, and measured at 593 nm. Results were expressed as Trolox equivalents (TE) per 100 mg FW.

### 2.9 Respiration Rate and External Ethylene Production

The respiration rate and ethylene production of tomatoes were measured to evaluate metabolic activity. Respiration rate (mL CO_2_ kg^–1^ h^–1^) was determined by enclosing fruits sealed jars and analyzing CO_2_ release with a headspace gas analyzer over 2 h. Ethylene production (μL C_2_H_4_ kg^–1^ h^–1^) was measured using by collecting gas samples and analyzed them via gas chromatography with flame ionization detection (GC-FID) after the incubation period, providing insights into ripening and physiological responses.^27^

### 2.10 Statistical Analysis

Statistical analysis was conducted to evaluate the effects of fertilizer applications and ripening stages. A two-factor factorial experiment was arranged in a randomized complete block design (RCBD) with 10 fertilizer treatments and 3 replications per treatment, resulting in a total of 30 plots. Each fertilizer combination was replicated equally. ANOVA was performed using SPSS 2020 software (Version 26), followed by Duncan’s Multiple Comparison Test for significant interactions.

To ensure the adequacy of the replication number relative to the treatment complexity, a priori power analysis was conducted using G*Power software. The analysis, based on a medium effect size (*f* = 0.25), α = 0.05, and sample size of 30, yielded a statistical power of 94%, indicating high sensitivity for detecting treatment and interaction effects.

The assumptions of normal data distribution and homogeneity of variances were verified. Principal Component Analysis (PCA) was conducted in XLSTAT to compare fertilizer effects and analyze parameter impacts.^28^ Prior to conducting PCA, all variables were standardized using z-score normalization to ensure comparability across different measurement scales. No log transformation was applied, as data distributions met normality assumptions. The variables included in the PCA were those presented in Tables 5 and 6, encompassing organic acids, phenolic compounds, antioxidant capacity, respiration rate, ethylene production, and carotenoid content (lycopene and β-carotene). A scree plot illustrating the eigenvalues of the principal components is provided as Figure 4 to support the selection of the first two components based on the Kaiser criterion (eigenvalue > 1.0). Sample adequacy (Kaiser-Meyer-Olkin, KMO = 0.760) and relationship strength (Bartlett’s test, *p* = 0.001) were confirmed in SPSS. Heat map clustering was performed in ClustVis^29^ to visualize variable relationships, identify patterns, and aid interpretation.

The Duncan multiple range test was employed to identify significant differences among group means following ANOVA. This test is commonly used in agricultural research due to its greater sensitivity in detecting differences, especially when multiple comparisons are involved.^30^ Although it is less conservative than Tukey’s HSD—controlling the Type I error rate per comparison rather than across the entire experiment—it remains widely accepted in exploratory studies, where biological variability is expected and detecting subtle treatment effects is important.

## 3 Results

### 3.1 Fruit Quality

Tomato fruit quality parameters including pH, titratable acidity (TA), total soluble solids (TSS), ascorbic acid (AsA), maturity index (MI), and flavor index (FI) were significantly influenced by fertilization treatments, maturity stages, and their interactions (Table 4). pH values ranged from 4.27 (T10, pink stage) to 4.59 (T10, green stage). Though pH did not significantly vary across fertilizer treatments alone, the interaction term (p^MxF^ = 0.0225) was significant. This suggests that pH is more sensitive to ripening stage than to fertilizer inputs, although some combinations (notably T3 and T6 at red stage) showed elevated pH values. Titratable acidity (TA) increased from green to pink stage and then declined by red maturity. T5 and T10 at pink stage had the highest TA (0.96%), while T7 at red stage had the lowest (0.46%). Fertilizer-induced acid metabolism shifts were most pronounced in combinations with vermicompost and organo-mineral fertilizers. Total soluble solids (TSS), a measure of sugar accumulation, increased with fruit maturity. The highest value (8.65 °Brix) was observed in T1 at red maturity, followed closely by T4. At green maturity, TSS values were consistently low across all treatments (<5.0 °Brix), reflecting the expected sugar accumulation timeline. Ascorbic acid (AsA) content, an important antioxidant parameter, ranged from 9.21 mg 100 g^–1^ (T8, green stage) to 52.32 mg 100 g^–1^ (T10, pink stage). Vermicompost-enriched treatments significantly increased AsA levels, especially at the red and pink stages notably T9 and T10, respectively. Maturity index (MI) values showed an increasing trend during ripening. The highest MI (16.99) was found in T1 at red maturity. T7 showed the lowest MI at the green stage (6.65), while pink-stage T2 (9.59) and T6 (8.20) displayed advanced ripening. Flavor index (FI), a combined metric of sweetness and acidity, varied significantly by treatment and stage. The highest FI (1.36) occurred in T1 at red maturity. At the pink stage, T5 (1.34), T10 (1.26), T3 and T4 (1.24) recorded superior values, suggesting enhanced sensory quality with specific fertilization strategies.

**Table 4:** Effects of Different Fertilization Combinations and Maturity Stages on the pH, Treatable Acidity (TA), Total Soluble Solids (TSS), Ascorbic Acid (AsA), Maturity Index (MI), and Flavor Index (FI) (Mean ± Standard Deviation)^a^

	treatments	pH	TA (%)	TSS (°Brix)	AsA (mg 100 g^–1^)	MI	FI
green maturity	T1	4.55 ± 0.13a-d	0.59 ± 0.09j-l	4.73 ± 0.15f	19.44 ± 1.14i-k	8.13 ± 1.46f-i	1.00 ± 0.02h
	T2	4.45 ± 0.05b-i	0.73 ± 0.06e-g	4.83 ± 0.15f	17.33 ± 2.01j-l	6.67 ± 0.73i	1.06 ± 0.02f-h
	T3	4.57 ± 0.05a-c	0.72 ± 0.04e-g	4.80 ± 0.10f	19.78 ± 1.01i-k	6.69 ± 0.52i	1.06 ± 0.01f-h
	T4	4.46 ± 0.12a-h	0.68 ± 0.03f-i	4.57 ± 0.25f	19.51 ± 0.12i-k	6.72 ± 0.61i	1.02 ± 0.01gh
	T5	4.54 ± 0.16a-e	0.69 ± 0.06f-h	4.83 ± 0.25f	13.10 ± 0.00l-n	7.03 ± 0.74hi	1.04 ± 0.02gh
	T6	4.57 ± 0.10a-c	0.65 ± 0.07g-k	4.73 ± 0.15f	16.03 ± 0.12k-m	7.35 ± 0.78hi	1.01 ± 0.03gh
	T7	4.44 ± 0.12c-i	0.74 ± 0.09d-f	4.87 ± 0.15f	19.51 ± 0.24i-k	6.65 ± 0.72i	1.07 ± 0.05f-h
	T8	4.49 ± 0.02a-h	0.64 ± 0.03g-k	4.80 ± 0.00f	9.21 ± 0.00n	7.46 ± 0.33hi	1.02 ± 0.01gh
	T9	4.54 ± 0.03a-d	0.68 ± 0.03f-i	4.63 ± 0.06f	12.14 ± 0.12mn	6.81 ± 0.42i	1.02 ± 0.01gh
	T10	4.59 ± 0.02a	0.65 ± 0.06g-k	4.73 ± 0.21f	13.10 ± 0.00l-n	7.32 ± 0.52hi	1.01 ± 0.04gh
pink maturity	T1	4.36 ± 0.01h-j	0.78 ± 0.01c-e	6.53 ± 0.06c-e	24.76 ± 0.41f-h	8.38 ± 0.09f-i	1.20 ± 0.01c-e
	T2	4.37 ± 0.02g-j	0.60 ± 0.01i-l	5.73 ± 0.06d-f	28.11 ± 1.74fg	9.59 ± 0.23e-h	1.08 ± 0.00e-h
	T3	4.32 ± 0.00ij	0.84 ± 0.04bc	6.67 ± 0.06c-e	42.43 ± 1.23 cd	7.99 ± 0.40f-i	1.24 ± 0.02a-d
	T4	4.39 ± 0.05g-j	0.87 ± 0.01b	6.33 ± 0.06c-e	46.46 ± 1.08bc	7.25 ± 0.00hi	1.24 ± 0.01a-d
	T5	4.38 ± 0.03g-j	0.96 ± 0.01a	7.30 ± 0.10bc	46.46 ± 0.74bc	7.62 ± 0.06g-i	1.34 ± 0.01ab
	T6	4.39 ± 0.01g-j	0.81 ± 0.10bd	6.60 ± 0.10c-e	48.09 ± 1.64ab	8.20 ± 0.96f-i	1.22 ± 0.05b-d
	T7	4.37 ± 0.01g-j	0.67 ± 0.04f-j	4.77 ± 0.15f	36.84 ± 1.48e	7.05 ± 0.23hi	1.03 ± 0.03gh
	T8	4.35 ± 0.00h-j	0.61 ± 0.05h-l	4.40 ± 0.00f	49.12 ± 2.16ab	7.22 ± 0.55hi	0.97 ± 0.02h
	T9	4.32 ± 0.01ij	0.67 ± 0.01f-j	4.67 ± 0.31f	48.44 ± 1.32ab	7.22 ± 0.53hi	1.03 ± 0.02gh
	T10	4.27 ± 0.02j	0.96 ± 0.04a	5.80 ± 0.10d-f	52.32 ± 0.43a	6.06 ± 0.28i	1.26 ± 0.02a-d
red maturity	T1	4.40 ± 0.06f-j	0.51 ± 0.01m-o	8.65 ± 0.35a	26.36 ± 1.22fg	16.99 ± 0.47a	1.36 ± 0.03a
	T2	4.53 ± 0.05a-f	0.54 ± 0.02l-n	5.37 ± 0.12ef	34.81 ± 1.15e	10.00 ± 0.53d-g	1.04 ± 0.01gh
	T3	4.58 ± 0.18ab	0.50 ± 0.01m-o	6.40 ± 1.78c-e	29.27 ± 5.44f	12.63 ± 3.15bc	1.14 ± 0.17d-g
	T4	4.42 ± 0.06d-i	0.67 ± 0.02f-j	8.33 ± 2.1ab	24.21 ± 1.61g-i	12.32 ± 2.75b-d	1.29 ± 0.16a-c
	T5	4.41 ± 0.08e-j	0.57 ± 0.01k-m	4.77 ± 0.21f	20.96 ± 1.79h-j	8.30 ± 0.36f-i	0.99 ± 0.02h
	T6	4.58 ± 0.05ab	0.48 ± 0.02no	4.53 ± 0.49f	36.63 ± 0.54e	9.57 ± 1.57e-h	0.96 ± 0.05h
	T7	4.47 ± 0.03a-h	0.46 ± 0.03o	6.67 ± 0.75c-e	38.35 ± 3.37de	14.40 ± 1.00b	1.18 ± 0.07c-f
	T8	4.51 ± 0.05a-g	0.58 ± 0.02k-m	6.83 ± 0.93 cd	21.58 ± 0.83h-j	11.84 ± 2.00c-e	1.17 ± 0.08c-f
	T9	4.44 ± 0.06c-i	0.63 ± 0.02h-k	7.37 ± 2.40a-c	49.14 ± 4.10ab	11.74 ± 3.71c-e	1.21 ± 0.19 cd
	T10	4.43 ± 0.03d-i	0.65 ± 0.01g-k	6.80 ± 1.22c-e	23.49 ± 10.41g-i	10.47 ± 1.81c-f	1.17 ± 0.09c-f
p^*Maturity (M)*^		<0.0001	<0.0001	<0.0001	<0.0001	<0.0001	<0.0001
p^*Fertilization (F)*^		0.1282	<0.0001	0.0016	<0.0001	<0.0001	<0.0001
p^*MxF*^		0.0225	<0.0001	<0.0001	<0.0001	<0.0001	<0.0001

a Values with the same letters in a column are not significantly different (pMxF, *p* < 0.05; Duncan’s test).T1: Control (no fertilizer); T2:100% CF; T3:100% VC; T4:100% OMF; T5:25% CF + 75% VC; T6:50% CF + 50% VC; T7:75% CF + 25% VC; T8:25% OMF + 75% VC; T9:50% OMF + 50% VC; T10:75% OMF + 25% VC.

### 3.2 Organic Acids

Organic acid composition was significantly affected by maturity stage (*p < 0.0001*), and all acids except succinic acid were significantly influenced by fertilizer treatments. However, significant M × F interactions were observed for all acids, including succinic acid (Table 5). Succinic acid was predominant at the green stage, especially in T6 (2811.98 μg g^–1^) and T7 (2679.96 μg g^–1^). This acid gradually declined at pink and red stages, giving way to citric and malic acids. Citric acid peaked at the pink stage in T4 (2871.47 μg g^–1^), indicating its metabolic dominance during midripening. Malic acid, associated with freshness and energy metabolism, was highest in T9 (1607.39 μg g^–1^) at red maturity. The malic/citric acid ratio varied across stages and treatments. Notably, T3 at the red stage displayed the highest ratio (1.253), suggesting a shift in organic acid synthesis or degradation. Tartaric acid appeared only at red maturity, with the highest level in T6 (15.99 μg g^–1^). These patterns reflect both developmental physiology and the modulatory effect of fertilizers, especially combinations involving VC and OMF.

**Table 5:** Effects of Different Fertilization Combinations and Maturity Stages on the Organic Acids (Mean ± Standard Deviation)^a^

maturity stage	fertilization	oxalic acid (μg g^–1^ FW)	citric acid (μg g^–1^ FW)	malic acid (μg g^–1^ FW)	succinic acid (μg g^–1^ FW)	fumaric acid (μg g^–1^ FW)	tartaric acid (μg g^–1^ FW)	malic/citric ratio
green maturity	T1	234.39 ± 98.21j	1295.04 ± 175.95c-e	646.49 ± 243.30g	2357.24 ± 168.25bc	93.14 ± 10.42b-h	-	0.490 ± 0.09kl
	T2	400.85 ± 98.66i	1273.46 ± 223.69c-e	863.94 ± 261.32e-g	2392.22 ± 137.48bc	83.11 ± 11.68e-i	-	0.713 ± 0.29f-k
	T3	475.71 ± 25.72b-i	1244.88 ± 118.51c-e	1213.46 ± 162.57a-f	2488.13 ± 219.07a-c	83.34 ± 12.14e-i	-	0.980 ± 0.05b-f
	T4	436.38 ± 52.80g-i	1901.93 ± 30.16b	1042.95 ± 353.89c-g	2332.41 ± 203.47bc	93.14 ± 15.66b-h	-	0.543 ± 0.17Cj-l
	T5	495.01 ± 3.11a-i	1530.77 ± 76.89c	1365.13 ± 26.31a-d	2421.56 ± 241.38a-c	83.81 ± 17.33e-i	-	0.893 ± 0.06c-h
	T6	541.43 ± 13.31a-e	1478.31 ± 134.06c	1487.69 ± 165.44ab	2811.98 ± 285.67a	82.04 ± 20.07e-i	-	1.013 ± 0.11a-e
	T7	539.62 ± 62.54a-g	1467.62 ± 123.36c	1502.58 ± 288.94ab	2679.96 ± 151.78ab	74.25 ± 14.68f-i	-	1.020 ± 0.05a-e
	T8	510.57 ± 8.83a-h	2067.41 ± 32.97b	1429.27 ± 59.54a-c	2396.73 ± 257.52bc	101.64 ± 24.14a-g	-	0.690 ± 0.01g-k
	T9	505.37 ± 12.81a-h	1425.99 ± 46.12 cd	1354.58 ± 84.69a-d	2101.09 ± 499.50c	75.43 ± 19.22e-i	-	0.950 ± 0.01b-g
	T10	460.29 ± 27.47d-i	1502.07 ± 115.10c	1190.66 ± 19.23a-f	2324.51 ± 208.57bc	66.69 ± 10.98g-i	-	0.800 ± 0.11e-j
pink maturity	T1	437.27 ± 25.31f-i	1327.23 ± 49.98c-e	938.94 ± 163.90d-g	1583.15 ± 61.43d	138.12 ± 5.53a	-	0.707 ± 0.10g-k
	T2	475.66 ± 38.20b-i	1420.40 ± 162.88 cd	1552.36 ± 599.93ab	1316.85 ± 77.41d-f	128.44 ± 8.20ab	-	1.083 ± 0.33a-d
	T3	491.07 ± 53.99a-i	1409.66 ± 17.98 cd	1191.19 ± 194.30a-f	982.84 ± 176.04f-i	95.26 ± 12.88b-h	-	0.843 ± 0.12d-i
	T4	510.36 ± 72.08a-h	2871.47 ± 229.22a	1162.87 ± 209.21b-f	1442.10 ± 230.76de	128.08 ± 36.15ab	-	0.403 ± 0.04l
	T5	464.87 ± 48.76c-i	1336.39 ± 171.93c-e	843.43 ± 154.13fg	763.93 ± 75.11h-j	72.36 ± 11.68f-i	-	0.637 ± 0.11h-l
	T6	446.44 ± 5.41e-i	1456.61 ± 76.04c	866.78 ± 168.30e-g	893.69 ± 246.42f-j	84.88 ± 21.12e-i	-	0.590 ± 0.08i-l
	T7	540.22 ± 31.60a-f	1397.04 ± 132.88 cd	1226.03 ± 228.66a-f	992.99 ± 192.49f-i	102.94 ± 24.11a-g	-	0.887 ± 0.21d-h
	T8	504.85 ± 13.79a-h	1008.04 ± 39.42e	1206.79 ± 31.63a-f	1071.98 ± 55.03e-i	102.23 ± 20.23a-g	-	1.197 ± 0.07ab
	T9	545.59 ± 21.91a-e	1320.36 ± 374.19c-e	1136.85 ± 182.20b-f	857.59 ± 354.79g-j	93.61 ± 23.95b-h	-	0.890 ± 0.17d-h
	T10	511.05 ± 11.28a-h	1233.53 ± 43.78c-e	1278.79 ± 46.79a-e	1321.36 ± 213.14d-f	110.02 ± 11.31a-f	-	1.036 ± 0.01a-e
red maturity	T1	480.97 ± 73.97a-i	1203.86 ± 88.47c-e	1255.57 ± 203.90a-f	534.86 ± 204.91j	56.78 ± 18.32h-i	5.58 ± 3.37b	1.036 ± 0.10a-e
	T2	514.64 ± 61.09a-h	1400.30 ± 164.40 cd	1281.26 ± 281.26a-e	1007.67 ± 212.06f-i	127.26 ± 20.55a-c	4.69 ± 1.11b	0.910 ± 0.11c-g
	T3	570.50 ± 34.17ab	1079.55 ± 100.20de	1356.14 ± 160.84a-d	1264.94 ± 266.64d-g	126.55 ± 25.64a-d	5.58 ± 1.78b	1.253 ± 0.05a
	T4	581.11 ± 52.13a	1231.76 ± 105.84c-e	1465.74 ± 117.75a-c	777.47 ± 94.07h-j	85.70 ± 10.90c-i	6.77 ± 2.56b	1.200 ± 0.19ab
	T5	466.12 ± 32.89c-i	1236.93 ± 65.64c-e	881.59 ± 52.81e-g	1187.04 ± 90.27d-h	85.23 ± 18.23d-i	6.03 ± 0.44b	0.713 ± 0.06f-k
	T6	464.63 ± 64.92c-i	1303.12 ± 108.62c-e	1135.68 ± 146.46b-f	1140.82 ± 219.93e-i	117.69 ± 19.30a-e	15.99 ± 6.64a	0.870 ± 0.06d-h
	T7	507.82 ± 57.47a-h	1346.05 ± 68.06c-e	1488.56 ± 120.23ab	752.65 ± 212.19h-j	103.06 ± 34.67a-g	2.57 ± 2.34b	1.107 ± 0.07a-d
	T8	565.65 ± 93.21a-c	1247.53 ± 202.39c-e	1150.95 ± 329.91b-f	706.38 ± 239.71ij	46.98 ± 23.78i	5.36 ± 3.12b	0.9133 ± 0.15c-g
	T9	554.83 ± 44.02a-d	1406.99 ± 210.55 cd	1607.39 ± 26.34a	1233.35 ± 408.20d-g	141.07 ± 54.52a	6.36 ± 2.56b	1.160 ± 0.18a-c
	T10	417.10 ± 73.11hi	1190.76 ± 155.80c-e	1204.47 ± 241.47a-f	732.33 ± 99.98ij	77.68 ± 12.23e-i	2.01 ± 0.44b	1.007 ± 0.09a-e
p^*Maturity (M)*^		0.0001	0.0001	0.0433	<0.0001	0.0008		<0.0001
p^*Fertilization (F)*^		<0.0001	0.0001	0.0009	0.4478	0.0470	0.0001	<0.0001
p^*MxF*^		0.0009	0.0001	<0.0001	<0.0001	<0.0001		<0.0001

a Values with the same letters in a column are not significantly different (pMxF, *p* < 0.05; Duncan’s test). T1: Control (no fertilizer); T2:100% CF; T3:100% VC; T4:100% OMF; T5:25% CF + 75% VC; T6:50% CF + 50% VC; T7:75% CF + 25% VC; T8:25% OMF + 75% VC; T9:50% OMF + 50% VC; T10:75% OMF + 25% VC.

### 3.3 Phenolics and Functional Compounds

As shown in Table 6, the maturity stage (M) significantly affected most phenolic and physiological parameters, while fertilization (F) had a selective impact most notably on gallic acid, hydroxycinnamic acid, total phenol (TP), respiration rate, and ethylene production. Significant M × F interactions were also observed for several variables, including gallic acid, rutin, ferulic acid, hydroxycinnamic acid, TAC, and ethylene, indicating stage specific and treatment specific effects.

**Table 6:** Effects of Different Fertilization Combinations and Maturity Stages on the Phenolics, Respiration Rate, and Ethylene Production (Mean ± Standard Deviation)^a^

maturity stage	fertilization	gallic acid (μg g^–1^ FW)	rutin (μg g^–1^ FW)	ferulic acid (μg g^–1^ FW)	hydroxycinnamic acid (μg g^–1^ FW)	quercetin (μg g^–1^ FW)	TP (mg 100 g^–1^ FW)	TAC (μmol TE g^–1^ FW)	respiration rate (mL CO_2_ kg^–1^ h^–1^)	ethylene production (μL kg^–1^ h^–1^)
green maturity	T1	8.85 ± 1.45g	47.71 ± 2.24a-c	4.28 ± 0.24a	4.38 ± 1.43a-c	-	12.77 ± 2.89	76.94 ± 30.92gh	34.51 ± 9.17	-
	T2	13.59 ± 0.71g	48.40 ± 3.83ab	3.92 ± 0.70ab	5.55 ± 1.33a	-	13.60 ± 1.57	75.14 ± 10.01h	25.95 ± 9.46	-
	T3	12.23 ± 1.03g	43.20 ± 3.09d-f	2.01 ± 0.94c-f	3.27 ± 0.83 cd	-	12.36 ± 2.27	74.31 ± 15.12h	25.54 ± 3.23	-
	T4	13.12 ± 0.69g	42.60 ± 2.17d-f	2.16 ± 0.19c-f	3.23 ± 0.39 cd	-	13.07 ± 2.08	71.25 ± 10.46h	23.20 ± 6.44	-
	T5	14.80 ± 1.43g	42.10 ± 2.85 d-f	2.39 ± 1.05b-f	2.95 ± 0.60 cd	-	14.74 ± 1.10	88.19 ± 8.27gh	22.04 ± 7.03	-
	T6	16.41 ± 3.18g	43.15 ± 3.97d-f	3.32 ± 2.41a-c	3.45 ± 1.09b-d	-	14.58 ± 0.64	95.28 ± 10.34gh	30.52 ± 12.84	-
	T7	13.57 ± 1.35g	42.89 ± 3.12d-f	3.02 ± 1.63a-f	3.35 ± 0.66 cd	-	13.54 ± 1.30	80.97 ± 17.56gh	29.87 ± 16.62	-
	T8	16.00 ± 0.30g	43.53 ± 3.24c-f	2.59 ± 0.42b-f	3.10 ± 0.20 cd	-	13.15 ± 1.14	67.36 ± 10.18h	25.03 ± 6.20	-
	T9	16.35 ± 0.07g	42.48 ± 2.98d-f	2.58 ± 1.22b-f	3.15 ± 0.63 cd	-	15.06 ± 1.85	90.56 ± 14.42gh	26.24 ± 9.09	-
	T10	14.56 ± 1.81g	48.59 ± 4.92a	3.16 ± 1.05a-e	3.85 ± 1.16b-d	-	13.19 ± 1.85	141.53 ± 121.50fg	26.78 ± 9.42	-
pink maturity	T1	29.55 ± 0.40f	41.49 ± 2.22d-f	1.84 ± 0.02c-f	3.07 ± 0.43 cd	-	19.01 ± 2.12	231.81 ± 48.99a-e	32.90 ± 0.75	5.75 ± 1.13a-c
	T2	31.99 ± 1.75f	39.83 ± 1.22ef	1.65 ± 0.48d-f	2.86 ± 0.43d	-	15.43 ± 1.50	164.72 ± 23.61ef	36.22 ± 0.14	5.90 ± 0.30ab
	T3	28.87 ± 2.04f	40.49 ± 0.80d-f	1.88 ± 0.31c-f	3.19 ± 0.26 cd	-	17.73 ± 2.56	196.67 ± 63.47d-f	39.57 ± 2.65	6.73 ± 0.56a
	T4	41.53 ± 1.21e	40.03 ± 0.19d-f	1.69 ± 0.05c-f	2.88 ± 0.23d	-	17.52 ± 1.02	195.97 ± 18.93d-f	33.67 ± 1.74	4.54 ± 0.81c-e
	T5	39.73 ± 2.15e	43.90 ± 0.88c-f	2.69 ± 0.61b-f	3.68 ± 0.37b-d	-	19.27 ± 1.53	207.78 ± 17.95c-f	31.76 ± 0.33	5.06 ± 1.40b-d
	T6	40.48 ± 2.74e	41.17 ± 0.40d-f	1.78 ± 0.49c-f	3.54 ± 0.15b-d	-	19.19 ± 1.97	206.67 ± 28.59c-f	25.79 ± 3.88	3.45 ± 0.21e-g
	T7	40.16 ± 2.68e	39.27 ± 0.52f	1.54 ± 0.28ef	2.95 ± 0.30 cd	-	17.71 ± 2.77	188.61 ± 25.19d-f	34.73 ± 2.98	6.57 ± 1.19a
	T8	45.19 ± 1.91de	44.56 ± 2.96a-d	3.09 ± 0.63a-f	4.40 ± 0.63a-c	-	20.02 ± 0.96	243.75 ± 28.80a-d	29.89 ± 1.41	3.95 ± 0.76d-f
	T9	50.48 ± 6.48 cd	44.54 ± 2.13a-d	2.26 ± 0.38c-f	3.67 ± 0.53b-d	-	20.16 ± 2.77	230.00 ± 19.61b-e	25.52 ± 1.99	4.09 ± 0.18d-f
	T10	40.13 ± 1.02e	44.23 ± 0.31b-e	1.88 ± 0.09c-f	3.17 ± 0.12bc	-	19.55 ± 1.64	214.31 ± 30.45c-e	28.86 ± 0.45	5.02 ± 0.92b-d
red maturity	T1	49.31 ± 3.24 cd	42.72 ± 1.26d-f	2.35 ± 0.37b-f	3.55 ± 0.34b-d	19.35 ± 1.85	21.38 ± 1.64	267.92 ± 33.80a-c	28.58 ± 6.40	4.08 ± 0.52d-f
	T2	53.05 ± 5.83a-d	41.96 ± 1.61d-f	2.84 ± 1.24a-f	3.82 ± 0.80ab	18.78 ± 1.98	20.25 ± 1.67	242.92 ± 22.85a-d	28.97 ± 2.71	2.06 ± 0.25g-i
	T3	54.68 ± 2.15a-c	42.31 ± 2.82d-f	2.29 ± 0.58c-f	3.38 ± 0.71 cd	17.49 ± 0.78	18.09 ± 1.65	201.39 ± 42.54c-f	34.27 ± 2.05	3.26 ± 0.10e-h
	T4	50.07 ± 7.04 cd	41.33 ± 2.10d-f	2.15 ± 0.69c-f	2.95 ± 0.30 cd	17.48 ± 1.52	20.96 ± 1.19	244.86 ± 20.84a-d	31.27 ± 4.33	2.92 ± 0.31f-i
	T5	54.53 ± 11.23a-c	40.02 ± 0.46d-f	1.50 ± 0.25f	3.10 ± 0.25 cd	16.90 ± 0.53	17.87 ± 1.05	193.47 ± 31.13d-f	30.12 ± 0.42	3.27 ± 0.61e-h
	T6	52.03 ± 12.89b-d	40.09 ± 1.28d-f	1.74 ± 0.47c-f	3.15 ± 0.18 cd	17.09 ± 0.85	20.45 ± 2.80	254.86 ± 43.79a-d	23.98 ± 4.67	1.81 ± 1.30i
	T7	58.50 ± 2.58ab	43.02 ± 2.42d-f	2.01 ± 0.27c-f	3.37 ± 0.67 cd	17.31 ± 0.63	21.47 ± 0.92	282.08 ± 32.81ab	25.82 ± 1.84	2.02 ± 0.60hi
	T8	50.12 ± 4.41 cd	40.64 ± 0.91d-f	1.99 ± 0.75c-f	3.65 ± 1.19b-d	19.24 ± 3.61	22.58 ± 2.09	250.14 ± 9.53a-d	19.38 ± 3.26	3.20 ± 0.74e-h
	T9	60.02 ± 2.28a	43.31 ± 1.71 d-f	3.20 ± 0.95a-d	4.83 ± 1.52ab	19.95 ± 1.72	22.66 ± 2.04	298.19 ± 21.88a	20.01 ± 3.50	2.96 ± 0.35f-i
	T10	49.62 ± 0.81 cd	39.78 ± 0.17ef	1.82 ± 0.17c-f	2.61 ± 0.00d	16.60 ± 0.00	17.91 ± 1.26	190.69 ± 19.85d-f	27.40 ± 1.16	3.24 ± 0.12e-h
p^*Maturity (M)*^		<0.0001	<0.0001	<0.0001	0.2933		<0.0001	<0.0001	0.0024	<0.0001
p^*Fertilization (F)*^		<0.0001	0.0523	0.2725	0.0466	0.1532	0.0151	0.0955	0.0353	<0.0001
p^*MxF*^		0.0045	0.0017	0.0461	0.0037		0.1082	0.0238	0.2549	<0.0001

a Values with the same letters in a column are not significantly different (pMxF, *p* < 0.05; Duncan’s test). T1: Control (no fertilizer); T2:100% CF; T3:100% VC; T4:100% OMF; T5:25% CF + 75% VC; T6:50% CF + 50% VC; T7:75% CF + 25% VC; T8:25% OMF + 75% VC; T9:50% OMF + 50% VC; T10:75% OMF + 25% VC.

Gallic acid consistently increased with ripening, peaking in T9 at the red stage (60.02 μg g^–1^), suggesting enhanced phenylpropanoid pathway activation under this treatment. Similarly, quercetin appeared only at full maturity, with its highest value also in T9, highlighting the treatment’s role in stimulating late-stage flavonol accumulation. Rutin showed a different trend highest at green stage (T10), declining thereafter while ferulic and hydroxycinnamic acids varied across stages and treatments without a consistent dominance.

Both TP and TAC increased during ripening, with red-stage T9 again demonstrating the highest values (22.66 mg 100 g^–1^ and 298.19 μmol TE g^–1^, respectively). However, notable TAC enhancements at the pink stage were also observed in T8 and T10, emphasizing that multiple OMF+VC-based combinations stimulated antioxidant capacity.

Physiological traits such as respiration and ethylene emission followed a climacteric pattern: ethylene production peaked at pink stage (T3), while respiration was highest in T3 and T7. These shifts coincided with secondary metabolite increases, implying a coordinated progression of ripening and bioactive compound biosynthesis.

Carotenoid accumulation further supported these trends. Lycopene and β-carotene increased markedly with maturity, reaching maximum levels in T9 at the red stage (41.15 and 14.41 μg g^–1^, respectively). Although T1 showed elevated carotenoid levels at the pink stage, the highest total pigment accumulation across all ripening stages was consistently observed in treatments receiving integrated fertilization (OMF+VC), highlighting their superior influence on lycopene and β-carotene synthesis (Figure 1).

**Figure 1:**
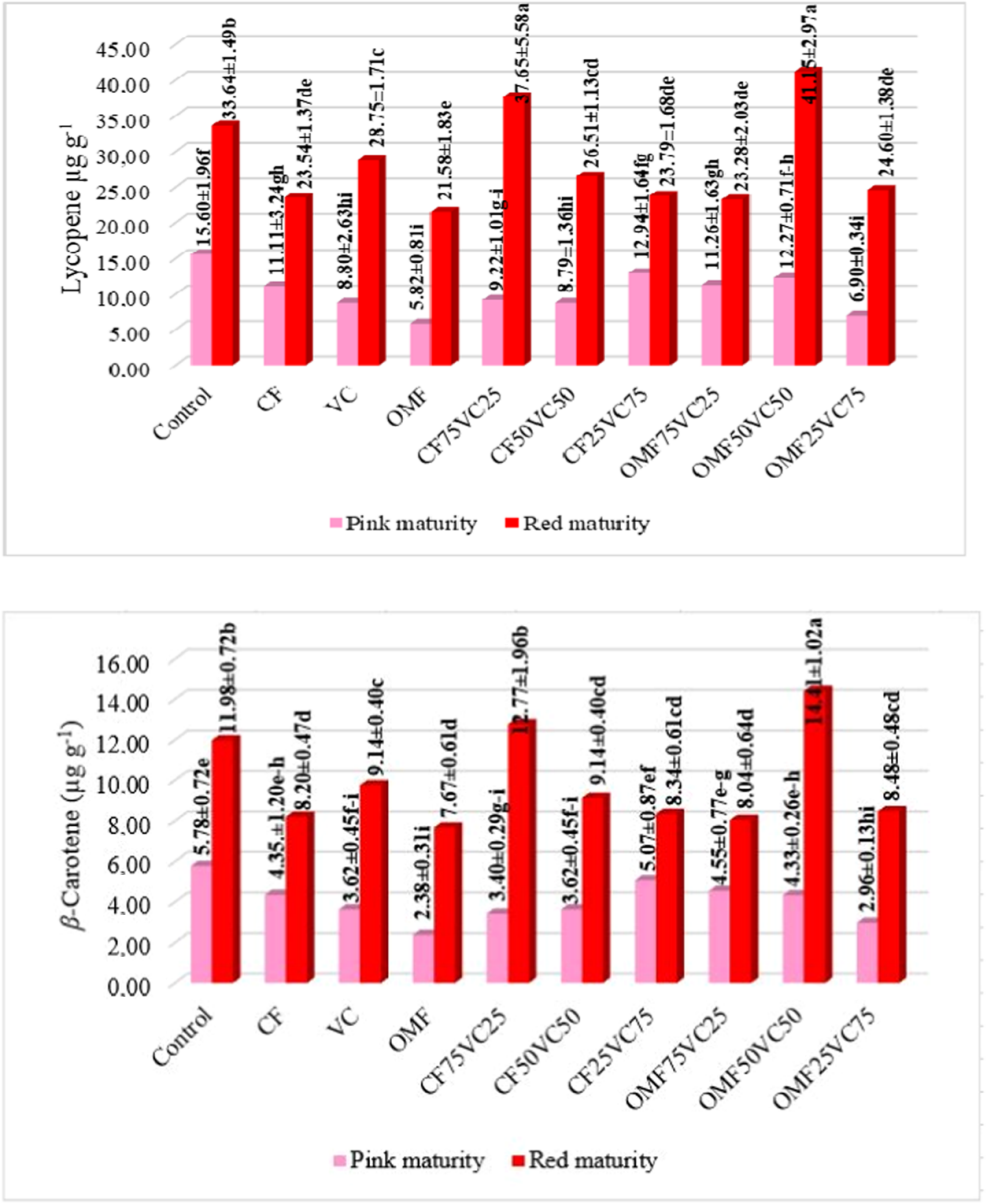
Changes in lycopene and β-carotene content (μg g^–1^ FW) of tomato fruits at pink and red maturity stages under different fertilization treatments. Data represent means ± standard deviation (SD) of three biological replicates (*n* = 3). Different letters indicate statistically significant differences among treatments within the same maturity stage (*p < 0.05*).P^*Maturity (M)*^ < 0.0001,P^*Fertilization (F)*^ < 0.0001,p^*MxF*^ < 0.0001. T1: Control (no fertilizer); T2:100% CF; T3:100% VC; T4:100% OMF; T5:25% CF + 75% VC; T6:50% CF + 50% VC; T7:75% CF + 25% VC; T8:25% OMF + 75% VC; T9:50% OMF + 50% VC; T10:75% OMF + 25% VC.

Taken together, the data suggest that OMF+VC applications not only enhanced individual compounds such as gallic acid, lycopene, and quercetin, but also supported the simultaneous activation of antioxidant systems, ripening physiology, and pigment biosynthesis. Rather than isolated improvements, these findings reflect a synergistic stimulation of metabolic pathways across developmental stages.

### 3.4 PCA and Cluster Analysis

Principal Component Analysis (PCA) revealed that the first two principal components explained substantial variance at each maturity stage (green: 51.99%, pink: 51.89%, red: 60.02%) (Tables 7–8, PCA plots). To justify component selection, a scree plot depicting eigenvalues up to PC6 is provided for each stage (Figure 2), with a combined plot (Figure 4) demonstrating a clear elbow point after PC2.

**Figure 2:**
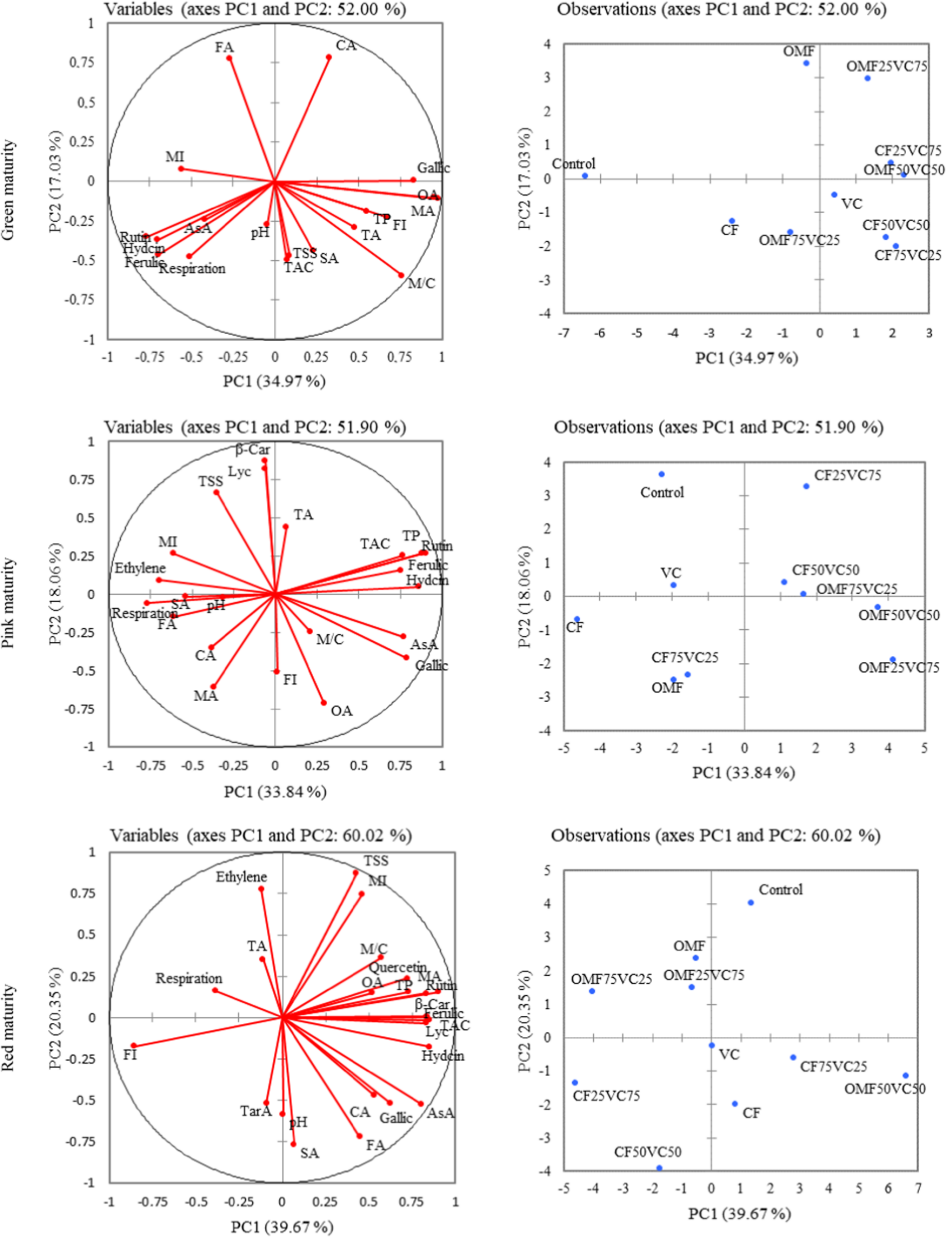
Loading plot conducted using characters and the score plot drawn according to the first two principle components of PCA analysis. T1: Control (no fertilizer); T2:100% CF; T3:100% VC; T4:100% OMF; T5:25% CF + 75% VC; T6:50% CF + 50% VC; T7:75% CF + 25% VC; T8:25% OMF + 75% VC; T9:50% OMF + 50% VC; T10:75% OMF + 25% VC.

**Table 7:** Principal Component Analysis Results Regarding Characters in the Tomato Fruits of Green Maturity Stage, Pink Maturity Stage^a^

green maturity stage	PC1	PC2	PC3	PC4	PC5	PC6	pink maturity stage	PC1	PC2	PC3	PC4	PC5	PC6							
eigenvalue	6.644	3.235	2.898	2.145	1.519	1.054	eigenvalue	7.444	3.973	3.557	2.148	1.892	1.061							
variability (%)	34.967	17.028	15.253	11.288	7.993	5.545	variability (%)	33.839	18.058	16.170	9.766	8.600	4.823							
cumulative Var. (%)	34.967	51.995	67.248	78.536	86.529	92.074	cumulative Var. (%)	33.839	51.896	68.067	77.832	86.432	91.256							
eigen vectors									eigen vectors											
OA	0.378	–0.059	0.005	0.030	–0.047	0.094	OA	0.106	–0.356	0.139	–0.272	–0.118	–0.273							
CA	0.126	0.434	–0.101	0.139	–0.182	0.149	CA	–0.141	–0.176	–0.358	–0.163	0.155	–0.007							
MA	0.368	–0.056	–0.083	0.154	0.016	0.105	MA	–0.137	–0.306	0.316	–0.080	–0.024	0.067							
SA	0.088	–0.242	0.130	0.460	0.114	0.179	SA	–0.197	–0.008	0.064	–0.157	0.545	0.256							
FA	–0.104	0.430	0.074	0.316	–0.052	0.101	FA	–0.225	–0.074	0.128	–0.069	0.512	0.124							
M/C	0.295	–0.331	0.021	0.023	0.148	0.015	M/C	0.076	–0.123	0.468	0.073	–0.060	0.171							
pH	–0.018	–0.150	–0.388	–0.119	0.459	0.185	pH	–0.116	–0.010	–0.298	0.457	0.008	–0.116							
TA	0.184	–0.160	0.453	–0.197	–0.086	0.030	TA	0.024	0.222	–0.289	–0.438	–0.093	0.175							
TSS	0.033	–0.258	0.190	0.210	–0.192	0.609	TSS	–0.129	0.332	–0.298	–0.091	–0.134	0.192							
AsA	–0.162	–0.134	0.384	0.076	0.365	–0.245	AsA	0.282	–0.140	–0.185	–0.210	–0.149	0.117							
gallic	0.322	0.004	–0.193	–0.049	–0.306	–0.024	Lyc	–0.023	0.411	0.208	–0.061	0.102	–0.358							
rutin	–0.297	–0.194	–0.098	–0.154	–0.270	0.224	*β-*Car	–0.022	0.438	0.197	–0.060	0.078	–0.235							
ferulic	–0.271	–0.257	–0.096	0.186	–0.337	–0.140	gallic	0.289	–0.209	–0.066	0.003	0.071	–0.345							
Hydcin	–0.272	–0.205	0.130	–0.094	–0.402	–0.015	rutin	0.321	0.134	0.128	–0.092	0.066	0.100							
TP	0.213	–0.104	–0.193	–0.052	–0.256	–0.459	ferulic	0.276	0.078	0.084	0.191	–0.087	0.378							
TAC	0.028	–0.274	–0.367	–0.290	0.036	0.150	Hydcin	0.315	0.024	0.047	0.274	–0.057	0.268							
respiration	–0.199	–0.265	–0.121	0.394	0.095	–0.264	TP	0.330	0.136	–0.027	–0.080	0.147	0.008							
MI	–0.218	0.045	–0.394	0.299	0.046	0.127	TAC	0.279	0.128	0.057	0.006	0.307	0.195							
FI	0.258	–0.126	–0.076	0.374	–0.135	–0.246	respiration	–0.282	–0.030	0.085	–0.062	–0.271	0.376							
							ethylene	–0.255	0.047	0.204	–0.187	–0.274	0.062							
							MI	–0.224	0.134	0.048	0.448	–0.043	–0.026							
							FI	0.004	–0.254	–0.225	0.179	0.213	0.072							

a OA: Oxalic acid, CA: citric acid, MA: malic acid, SA: succinic acid, FA: fumaric acid, M/C: malic/citric ratio, TA: titratable acidity, TSS: total soluble solids, AsA: ascorbic acid, Hydcin: hydroxycinnamic acid, TP: total phenol, TAC: total antioxidant capacity, MI: maturity index, FI: flavor index.

**Table 8:** Principal Component Analysis Results Regarding Characters in the Tomato Fruits of Red Maturity Stage^a^

red maturity stage	PC1	PC2	PC3	PC4	PC5	PC6
eigenvalue	9.522	4.884	2.826	1.767	1.721	1.273
variability (%)	39.673	20.348	11.774	7.362	7.169	5.305
cumulative Var. (%)	39.673	60.021	71.795	79.158	86.327	91.632
eigen vectors						
OA	0.168	0.067	0.179	0.435	–0.165	0.097
CA	0.172	–0.213	–0.309	0.078	0.153	–0.254
MA	0.270	0.066	0.181	0.090	0.032	–0.346
SA	0.022	–0.346	0.225	0.114	0.169	0.335
FA	0.145	–0.326	0.270	0.056	0.121	–0.037
TarA	–0.029	–0.233	–0.115	0.104	–0.381	0.120
M/A	0.185	0.164	0.368	0.067	–0.053	–0.209
pH	0.001	–0.263	0.191	0.148	–0.466	0.113
TA	–0.036	0.160	–0.058	0.453	0.475	–0.135
TSS	0.139	0.394	0.033	0.044	0.019	–0.119
AsA	0.260	–0.237	–0.002	–0.080	0.058	–0.107
Lyc	0.270	–0.014	–0.028	–0.376	0.074	0.085
β-Car	0.272	0.001	–0.045	–0.363	0.088	0.102
gallic	0.202	–0.234	0.103	–0.198	0.273	0.024
rutin	0.293	0.071	0.157	–0.148	–0.024	0.040
ferulic	0.270	0.003	0.073	0.238	0.132	0.120
Hydcin	0.275	–0.080	–0.110	0.132	0.111	0.348
quercetin	0.234	0.108	–0.206	0.207	–0.034	0.377
TP	0.235	0.071	–0.323	0.152	–0.209	–0.092
TAC	0.275	–0.007	–0.244	–0.041	–0.169	–0.153
respiration	–0.125	0.074	0.465	–0.083	–0.002	–0.012
ethylene	–0.039	0.350	0.036	–0.057	0.172	0.486
MI	0.150	0.338	0.056	–0.187	–0.268	0.094
FI	–0.277	–0.078	–0.211	–0.092	0.137	0.057

a OA: oxalic acid, CA: citric acid, MA: malic acid, SA: succinic acid, FA: fumaric acid, TarA: tartaric acid, M/C: malic/citric ratio, TA: titratable acidity, TSS: total soluble solids, AsA: ascorbic acid, Lyc: lycopene, β-Car: β-carotene, Hydcin: hydroxycinnamic acid, TP: total phenol, TAC: total antioxidant capacity, MI: maturity index, FI: flavor index.

At the green stage, PC1 was predominantly shaped by high positive loadings from oxalic acid (0.378), malic acid (0.368), and gallic acid (0.322), while PC2 was driven by citric (0.434) and fumaric acids (0.430), indicating that early stage acid metabolism was a major differentiating factor.

At the pink stage, PC1 had high contributions from ascorbic acid (0.282), rutin (0.321), total phenolics (0.330), and hydroxycinnamic acid (0.315), reflecting antioxidant accumulation and secondary metabolism. PC2, on the other hand, was positively influenced by lycopene (0.411), β-carotene (0.438), and flavor index (−0.254), linking it to functional and sensory quality.

In red maturity, PC1 was strongly associated with antioxidants and pigments, such as rutin (0.293), ferulic acid (0.270), lycopene (0.270), and TAC (0.275), indicating coordinated biosynthetic responses. Meanwhile, PC2 featured significant contributions from succinic acid (−0.346), ethylene (0.350), and maturity index (0.338), capturing variation in ripening behavior and physiological development.

PCA score plots (Figure 2) clearly separated treatment groups, with T9, T3, and T4 frequently clustering in regions defined by high-quality biochemical attributes. Heat map analysis (Figure 3) further confirmed these groupings, clustering traits such as TP, TAC, AsA, carotenoids, and FI especially at the red stage—suggesting a coherent pattern of quality enhancement through OMF+VC treatments.

**Figure 3:**
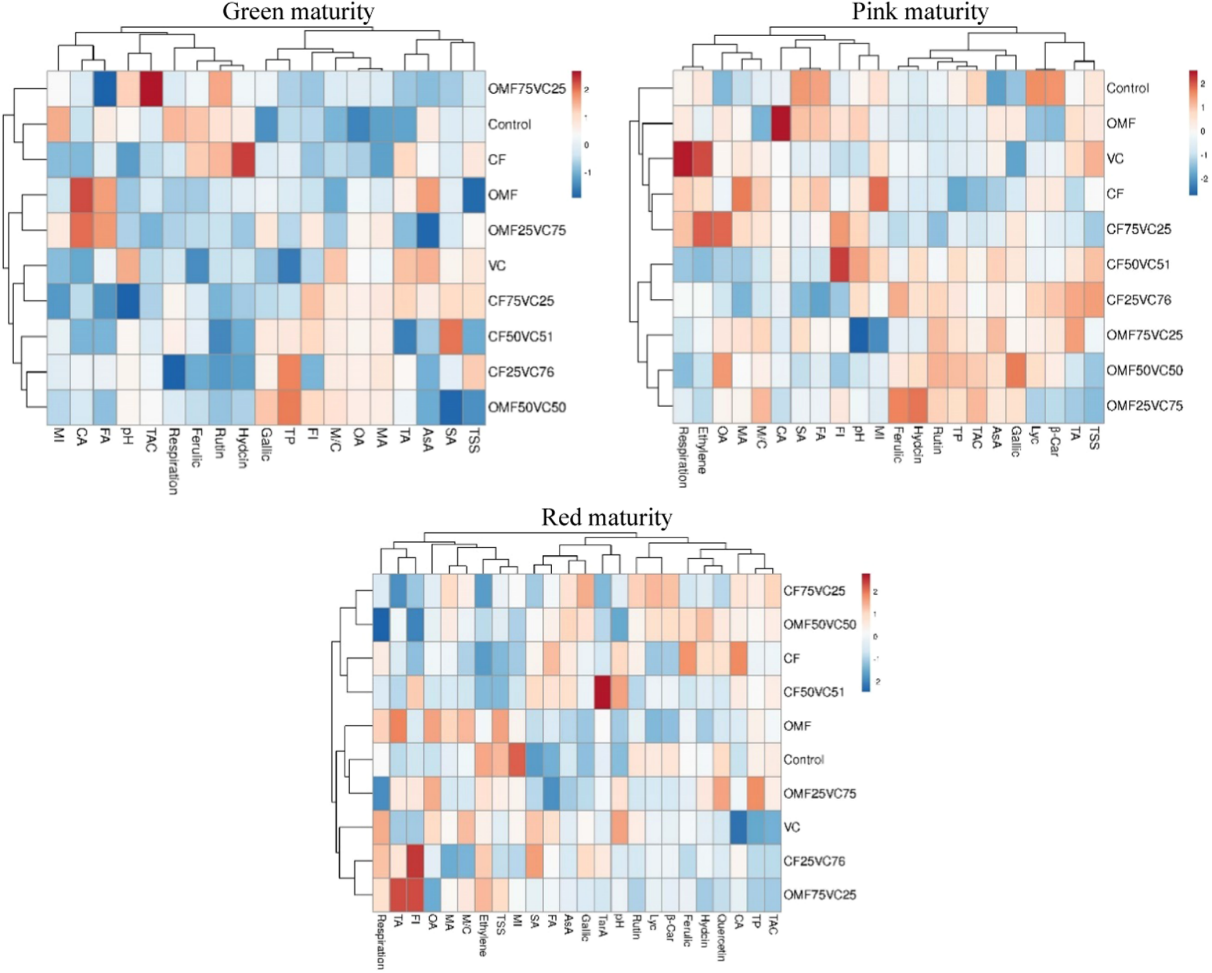
Hierarchical cluster heat map of effects of different fertilization applications on fruit quality in three maturity stages. OA: oxalic acid, CA: citric acid, MA: malic acid, SA: succinic acid, FA: fumaric acid, TarA: tartaric acid, M/C: malic/citric ratio, TA: titratable acidity, TSS: total soluble solids, AsA: ascorbic acid, Lyc: lycopene, β-Car: β-carotene, Hydcin: hydroxycinnamic acid, TP: total phenol, TAC: total antioxidant capacity, MI: maturity index, FI: flavor index- T1: Control (no fertilizer); T2:100% CF; T3:100% VC; T4:100% OMF; T5:25% CF + 75% VC; T6:50% CF + 50% VC; T7:75% CF + 25% VC; T8:25% OMF + 75% VC; T9:50% OMF + 50% VC; T10:75% OMF + 25% VC.

## 4 Discussion

The present study provides comprehensive evidence on how different fertilizer strategies, particularly combinations involving chemical fertilizers (CF), organo-mineral fertilizers (OMF), and vermicompost (VC) affect the biochemical and physiological characteristics of tomato fruits at different ripening stages. The results confirm and expand upon previous literature while offering practical insights for sustainable tomato production.

### 4.1 Influence of Fertilization on Fruit Quality

Tomato fruit pH values were generally within the safety threshold of 4.4, as emphasized by Teka,^31^ which is crucial for microbial stability. Brown, Lopes Sobrinho et al.^32,33^ noted that pH not only determines microbial safety but also significantly contributes to flavor, with lower pH indicating sourness and higher values suggesting sweetness. Our findings indicate that treatments involving vermicompost (e.g., T10) helped stabilize or slightly elevate pH levels, particularly at the pink stage. This corroborates with Truong et al.,^34^ who reported minimal effect of OMFs on pH, reinforcing the idea that pH is modulated more by ripening stage than by fertilization type. Titratable acidity (TA) and total soluble solids (TSS), as essential flavor components, exhibited dynamic changes during ripening, consistent with sugar biosynthesis and polysaccharide breakdown, as reported by Teka^31^ and Tran et al.^35^ As ripening progressed, TA decreased while TSS increased (Table 4). In line with Zhang et al.,^36^ our findings underscore the critical role of the TA/TSS balance in determining flavor quality. Previous studies by Lopes Sobrinho et al.^33^ and Truong et al.^34^ have highlighted the positive effects of organic amendments in enhancing TSS levels. In our study, T5 and T10 exhibited notably higher TA values at the pink maturity stage, demonstrating the advantage of integrated fertilization during intermediate ripening phases.

Ascorbic acid (AsA) content increased significantly through ripening, peaking at pink and red stages depending on treatment (Table 4). These patterns were consistent with previous reports by Du̅ma et al.^37^ and Ayuso-Yuste et al.^38^ The highest AsA content observed in T10 and T9 aligns with the findings of Truong et al.^34^ and Carricondo-Martínez et al.,^39^ who emphasized the role of VC in boosting antioxidant capacity. Gallie^40^ noted that AsA biosynthesis is genotype- and environment-dependent, and our findings further validate this in the context of fertilizer-induced stress modulation. Interestingly, Olowokere^41^ also observed elevated AsA levels under CF treatments, suggesting that both organic and chemical inputs can modulate AsA depending on stage and dose.

The maturity index (MI) and flavor index (FI) are composite indicators of consumer-preferred quality. Zhu et al.^42^ identified MI as an effective marker of sensory balance. Our results confirmed this, with MI peaking at red maturity and being modulated significantly by fertilization strategy (Table 4). Navez et al.^18^ stated that FI values above 0.85 indicate acceptable taste; all our treatments met or exceeded this value. Hernández Suárez et al.^43^ and Murariu et al.^44^ reported lower FI values in open-field or low-input systems, suggesting our input strategies enhanced both biochemical and sensory attributes.

The role of fertilization in modulating lycopene synthesis was emphasized by Waliszewski and Blasco^45^ and Fortis-Hernández et al.,^46^ who described the stress-induced enhancement of lycopene in response to organic input. Arias et al.^47^ and Radzevičius et al.^48^ linked lycopene accumulation to the chloroplast-to-chromoplast transition, a process active during ripening. Our PCA results confirmed lycopene’s association with red maturity and phenolic synthesis, echoing Imran et al.^49^ and Farneti et al.,^50^ who noted lycopene’s dominant role in antioxidant protection.

### 4.2 Influence of Fertilization on Organic Acids

Organic acid composition changed significantly with stage and treatment (Table 5). Succinic acid predominated at green maturity, followed by citric and malic acids. This partially aligns with Erdinc et al.,^51^ who identified citric and succinic acids as dominant, though in our study, succinic acid was more prominent at earlier stages, and citric acid dominated later. The dominance of citric and malic acids during ripening matches findings from Petropoulos et al.,^52,53^ Batista-Silva et al.,^54^ and Li et al.^55^ The malic/citric ratio, an indicator of freshness,^56,57^ exceeded 1.0 in certain treatments (e.g., T3), suggesting altered acid metabolism under organic or mixed inputs. The appearance of tartaric acid only at red maturity reflects secondary metabolism enhancement with ripening and stress. Hallmann,^58^ Pieper and Barrett^59^ emphasized that organic inputs generally increase organic acid accumulation, supporting our observations.

Beyond the major organic acids (succinic, citric, and malic), oxalic and fumaric acids exhibited distinct patterns across maturity stages and treatments. Oxalic acid, often associated with calcium regulation and plant stress responses,^53^ was highest at green and pink stages, particularly in T6, T7, and T9, reflecting early stage metabolic demands and the influence of organic inputs. This suggests that fertilization strategies integrating vermicompost or organo-mineral combinations might modulate oxalic acid metabolism under stress conditions.

Fumaric acid, a key intermediate in the tricarboxylic acid (TCA) cycle, exhibited a progressive increase across ripening stages, peaking at the red maturity stage in T9 (141.07 μg g^–1^). This pattern supports its role in sustaining energy metabolism and carbon balance during advanced ripening, contributing to flavor development and fruit softening.

The appearance of tartaric acid exclusively at red maturity, particularly in T6, may indicate the activation of secondary metabolic pathways related to flavor enhancement or stress mitigation in later stages of ripening.^54^ Tartaric acid accumulation, though less commonly discussed in tomatoes, may contribute to acid balance and taste complexity, supporting a broader biochemical diversification during late ripening.

These findings align with Hallmann^58^ and Pieper and Barrett^59^ and, who reported that organic fertilization strategies often enhance organic acid diversity and accumulation, promoting better flavor profiles and postharvest stability. Collectively, the dynamic shifts in oxalic, fumaric, and tartaric acids, influenced by fertilization and ripening stage, highlight the intricate regulation of primary and secondary metabolism in tomato fruits, shaped by environmental inputs and developmental cues.

### 4.3 Influence of Fertilization on Phenolics and Functional Compounds

Phenolic compounds, including gallic acid, rutin, ferulic acid, hydroxycinnamic acid, and quercetin, varied significantly across maturity stages and fertilization treatments (Table 6). Gallic acid and rutin were identified as the dominant phenolic compounds in this study. In contrast, Li et al.^55^ and Erdinc et al.^51^ reported rutin and chlorogenic acid as the predominant phenolics in tomatoes. While chlorogenic acid was not analyzed in the present study, the dominance of gallic acid highlights potential differences due to cultivar, fertilization strategy, or environmental conditions. Gallic acid significantly increased during ripening, contrary to Tao et al.,^60^ who reported a decrease. This discrepancy may stem from cultivar or environmental interactions. Buta and Spaulding^61^ observed a decline in rutin postanthesis, also evident in our findings. Quercetin, detected only at red maturity, aligns with Slimestad and Verheul,^62^ who noted its late-stage biosynthesis. Anton et al.^63^ and Kosem et al.^64^ reported that organic inputs stimulate flavonoid synthesis, a phenomenon attributed to the presence of humic substances and hormone-like compounds in VC, as described by Panuccio et al.^65^ The enhanced antioxidant and phenolic content in OMF+VC treatments may stem from the synergistic effects of organic matter, humic acids, and slow-release nutrients. These components potentially activate secondary metabolic pathways under moderate stress conditions. Phenolic compounds such as gallic acid and rutin play crucial roles in detoxifying reactive oxygen species and contribute to dietary fiber and human health benefits.^66,67^

The enhanced phenolic content, lycopene accumulation, and improved flavor indices observed in OMF+VC treatments are likely mediated by multiple interacting physiological mechanisms. Vermicompost is a rich source of humic and fulvic acids, plant growth hormones (e.g., auxins, cytokinins), and beneficial microorganisms, which enhance root development and nutrient uptake while improving stress resilience.^68,69^ When combined with organo-mineral fertilizers that supply macro- and micronutrients in balanced proportions, these inputs enhance metabolic activity within the rhizosphere. Phenolic biosynthesis is controlled by environmental cues and nutrient availability. Organic inputs can impose moderate stress and provide sustained nutrient release, activating the phenylpropanoid pathway and increasing levels of gallic acid, rutin, and other antioxidant phenolics. Likewise, lycopene biosynthesis during ripening is triggered by oxidative stress and plastid transitions mechanisms that may be modulated by vermicompost-derived humic substances and nutrient signals. Collectively, these findings align with reports that organic fertilization significantly enhances secondary metabolite accumulation, such as increases in TSS, vitamin C, lycopene and overall fruit quality in tomatoes.^70,71^ Therefore, the synergy between OMF and VC supports plant nutrition and stimulates key biosynthetic pathways, resulting in improved biochemical quality and flavor in tomato fruits.

The total phenolic (TP) content and total antioxidant capacity (TAC) varied significantly with maturity stages and fertilization treatments (Table 8). At green maturity, TP content remained consistent (12.36–15.06 mg 100 g^–1^), while TAC was highest in T10 (141.53 μmol TE g^–1^). At pink maturity, TP increased across treatments, peaking in T9 (20.16 mg 100 g^–1^), while TAC was highest in T8 (243.75 μmol TE g^–1^). At red maturity, TP content reached its highest in T9 (22.66 mg 100 g^–1^), with TAC peaking at 298.19 μmol TE g^–1^. These findings emphasize the importance of selecting appropriate fertilization strategies to optimize tomato quality across maturity stages. Fortis-Hernández et al.^72^ reported TP ranging from 36.0 to 56.94 mg 100 g^–1^, with mineralized compost increasing TP by 56% compared to vermicompost. Naguib et al.^73^ highlighted the stimulating effect of organic fertilization on phenolic synthesis, enhancing antioxidant activity, consistent with Li et al.^55^ Du̅ma et al.^37^ found a decrease in TP with ripening, while García-Valverde et al.^74^ reported higher TP at green and midripening stages. Raffo et al.^75^ and Ayuso-Yuste et al.^38^ observed high TAC at the pink stage, with increases through ripening.

Carotenoids such as lycopene and β-carotene were also highly responsive to fertilizer types, especially at red maturity (Figure 1). Murmu et al.^76^ observed higher lycopene with combined CF and VC inputs. Carricondo-Martínez et al.^39^ found β-carotene favored by organic fertilization, while lycopene was more responsive to chemical inputs. Our data aligned with this trend, showing T9, an integrated treatment, as the most effective. Borguini et al.^77^ reported negligible differences between organic and conventional inputs, which contrasts with our findings, possibly due to cultivar or climatic differences.

Respiration rate and ethylene production, key indicators of ripening physiology, were also influenced by fertilization strategies and ripening stages. The T3 treatment had the highest respiration rates, while T9 and T8 showed the lowest at pink and red stages, respectively. Consistent with Zhu et al.^42^ and Ustun et al.,^78^ respiration rate increased at the turning stage, peaking during the climacteric phase. Ugur et al.^79^ linked higher respiration rates at green maturity to rapid fruit ripening. Ethylene production peaked at the pink maturity stage, especially in T3, and declined at red maturity, with the lowest levels observed in T6 and T8. No ethylene was produced at green maturity. This aligns with Batu,^80^ who also reported the highest ethylene production at pink maturity. These patterns confirm that fertilization modulates ripening dynamics, influencing shelf life and fruit quality.

### 4.4 PCA and Cluster Interpretation

Principal Component Analysis (PCA) was used to assess the impact of fertilizer combinations on tomato fruits at various maturity stages (Tables 7–8, Figure 2). Multivariate analyses confirmed the interdependence of traits and the grouping of treatments with similar quality profiles. Mozafari et al.^81^ and Seymen et al.^82^ emphasize that when the first two principal components explain more than 25% of the total variance as was the case in our PCA reliable interpretation of complex biochemical data is ensured. In this study, PC1 and PC2 explained over 50% of the variability at each ripening stage, confirming the robustness of the PCA model. To justify the component selection, a scree plot displaying the eigenvalues for the first six principal components at each maturity stage is presented (Figure 4). The clear inflection point after PC2 confirms the suitability of retaining the first two components for multivariate interpretation.

**Figure 4:**
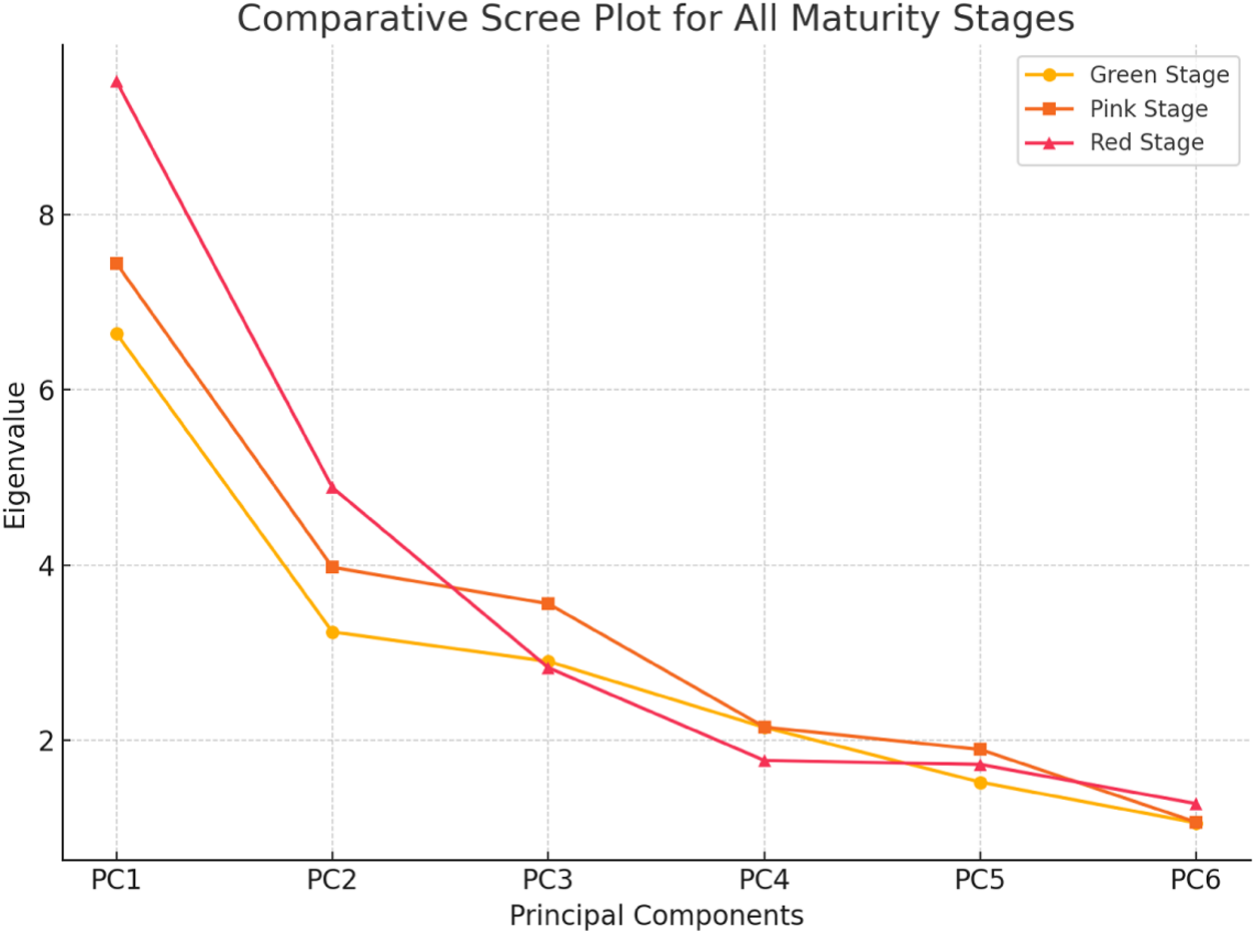
Combined scree plot displaying the first six principal components across tomato maturity stages (green, pink, red).

The PCA loading plots (Figure 2) revealed strong positive correlations between carotenoids (*lycopene*, *β-carotene*), phenolic compounds (gallic acid, rutin, and ferulic acid), and antioxidant capacity (TAC and TP), suggesting a shared biosynthetic or regulatory pathway. This biochemical synergy supports the concept of interconnected metabolic responses to fertilization and ripening stages, as described by Fortis-Hernandez et al.^46^ and Petropoulos et al.^52^ In particular, antioxidant compounds clustered with flavor-related attributes such as TSS, MI, and FI, indicating that both nutritional and sensory qualities can be optimized together.

The observation plots (Figure 2) clearly separated treatments based on their performance. T1 consistently appeared as an outlier, while T9, T4, and T6 were grouped as high-performing treatments, especially at the red maturity stage. These treatments, mostly composed of OMF+VC combinations, showed favorable profiles across multiple quality parameters. This aligns with previous findings^37,62^ suggesting that organic-based fertilization enhances the accumulation of quality-related secondary metabolites due to improved nutrient uptake and phytohormonal stimulation.

From a practical perspective, such treatment clusters offer actionable recommendations for growers. T9, in particular, stood out with a balanced and consistent enhancement in antioxidant compounds, flavor indices, and carotenoids across all stages. This supports its potential as a strategic fertilization choice for premium tomato production, with benefits extending to shelf life and consumer appeal. Cluster heat maps (Figure 3) further supported these findings by grouping traits and treatments with similar responses, highlighting the integrative effects of nutrient sources on tomato fruit quality.

While the findings provide valuable insights into the effects of OMF+VC on tomato fruit quality, the experiment was conducted at a single location under specific climatic and soil conditions. Therefore, generalization to other agroecological zones requires caution. Moreover, seasonal fluctuations in temperature and solar radiation may influence biochemical responses, especially in open-field systems. Future multilocation trials and longer-term studies are recommended to validate the reproducibility of results. Additionally, although OMF+VC combinations enhanced fruit quality, their economic feasibility for smallholder farmers must be evaluated through cost-benefit analyses.

## 5 Conclusions

This study demonstrated that the biochemical properties of tomato fruits at different ripening stages were significantly influenced by the applied fertilizer combinations. In particular, the application of organo-mineral fertilizers in combination with vermicompost (T9 and T10) led to notable increases in ascorbic acid, phenolic compounds, antioxidant capacity, and carotenoid content at the red maturity stage.

The findings indicate that integrated fertilization strategies are not only effective in enhancing fruit quality but also in reducing the reliance on chemical fertilizers. In this respect, the study offers environmentally friendly alternatives for sustainable tomato production and provides practical recommendations for growers.

Moreover, these results should be validated under different geographical conditions, soil types, and tomato cultivars. Long-term field trials are also necessary to evaluate the effects of these fertilizer combinations on soil health and microbial activity. This would further support environmentally responsible and consumer-oriented tomato production.

Accordingly, the informed use of organic and organo-mineral fertilizers may present an effective approach in addressing climate change and agricultural environmental pollution.

## Supplementary Material



## Notes

ANOVA results including *F*-values, degrees of freedom (df) for all measured parameters at different maturity stages and fertigation treatments (Table S1) (PDF)
