# Supplementary material for: Influence of Combinations of Chemical and Organic Fertilizers on Biochemical Responses of Tomato Fruits in Different Maturity Stages
Source: ACS Omega. 2025 Jul 14;10(28):30599–615. doi: 10.1021/acsomega.5c02531 (PMC12290969; doi:10.1021/acsomega.5c02531)
Supplement: Supplementary file 1 [file ao5c02531_si_002.pdf]

Influence of Combinations of Chemical and Organic Fertilizers on Biochemical Responses of Tomato Fruits in Different Maturity Stages

Aytekin EKINCIALP[1], Selma KIPCAK BITIK[1*], Çeknas ERDINÇ[2], Suat SENSOY[3]

[1]Van Yuzuncu Yil University, Baskale Vocational School, 65080 Van, Türkiye

[2]Van Yuzuncu Yil University, Faculty of Agriculture, Department of Agricultural Biotechnology, 65080 Van, Türkiye

[3]Van Yuzuncu Yil University, Faculty of Agriculture, Department of Horticulture, 65080 Van, Türkiye

*: Corresponding author: selmakipcak@yyu.edu.tr

Contents

ANOVA results including F-values, degrees of freedom (df) for all measured parameters at different maturity stages and fertigation treatments.

Table S1. Supplementary Table: ANOVA Results with F-values and Degrees of Freedom (df)

Table S1. Supplementary Table: ANOVA Results with F-values and Degrees of Freedom (df)

Parameter	Maturity F-value	Maturity df	Fertigation F-value	Fertigation df	Maturity × Fertigation F-value	Maturity × Fertigation df
OA	7.88	2	7.0	9	3.59	18
CA	18.23	2	13.38	9	9.86	18
MA	3.31	2	3.74	9	3.65	18
SA	390.07	2	1.0	9	4.88	18
FA	8.06	2	2.07	9	4.04	18
TarA	125.38	2	4.91	9	4.91	18
M/C	21.18	2	6.66	9	6.74	18
pH	46.06	2	1.63	9	2.02	18
TSS	44.25	2	3.48	9	5.81	18
AsA	785.97	2	19.62	9	31.8	18
Gallic	683.25	2	6.53	9	2.48	18
Rutin	14.18	2	1.91	9	2.76	18
Ferulic	10.9	2	1.27	9	1.8	18
Hydcin	1.25	2	2.07	9	2.54	18
Quercetin	3519.95	2	1.55	9	1.55	18
TP	109.27	2	2.55	9	1.54	18
TAC	164.16	2	1.76	9	2.0	18
Respiration	6.67	2	2.19	9	1.25	18
Ethylene	547.43	2	5.34	9	4.65	18
TA	181.49	2	14.33	9	12.28	18
MI	118.3	2	4.87	9	4.17	18
FI	33.6	2	4.88	9	7.89	18
Lyc	2034.7	2	22.6	9	15.2	18
β-Car	1786.53	2	18.64	9	14.02	18

OA: Oxalic acid, CA: Citric acid, MA: Malic acid, SA: Succinic acid, FA: Fumaric acid, TarA: Tartaric acid, M/C: Malic/citric ratio, TA: Titratable acidity, TSS: Total soluble solids, AsA: Ascorbic acid, Lyc: Lycopene, β-Car: β-Carotene, Hydcin: Hydroxycinnamic acid, TP: Total phenol, TAC: Total antioxidant capacity, MI: Maturity index, FI: Flavor index.
